# Between the personal and the political: life experiences during the covid-19 pandemic in Tucumán, Argentina, 2020-2022

**DOI:** 10.1590/S0104-59702023000100030

**Published:** 2023-08-14

**Authors:** María Laura Cordero, Eric D. Carter

**Affiliations:** i Investigadora Asistente, Consejo Nacional de Investigaciones Científicas y Técnicas (Conicet)/Instituto de investigaciones territoriales y tecnológicas para la producción del hábitat/Universidad Nacional de Tucumán.San Miguel de Tucumán – Tucumán – Argentina lauracordero@conicet.gov.ar; ii Profesor titular, Macalester College.Saint Paul – MN – USA ecarter@macalester.edu

**Keywords:** Pandemic, Covid-19, Mixed methods, Symbolic frames, Argentina, Pandemia, Covid-19, Métodos mixtos, Marco simbólico, Argentina

## Abstract

This article analyzes feelings, experiences, practices, and actions that underlie the meanings attributed to the covid-19 pandemic. Based on a case study located in the province of Tucumán (Argentina), a mixed-methods investigation was developed, interested in capturing life experiences. Discourse analysis show the resignification of life itself, the valorization of close ties, community social capital, the State and politics. From the personal to the political, the interpretive frames people use to signify life experiences during the covid-19 pandemic exhibit differentiated feelings, experiences, practices, and actions.

## Resumen

Este artículo analiza sentimientos, experiencias, prácticas y acciones que subyacen a los significados atribuidos a la pandemia por covid-19. A partir de un estudio de caso situado en la provincia de Tucumán (Argentina), se desarrolló una investigación mixta, interesada en captar experiencias de vida. Los discursos evidencian la resignificación de la propia vida, la valorización de los vínculos, el capital social comunitario, el Estado y la política. Desde lo personal o desde lo político, el marco interpretativo con el que las personas significan las experiencias de vida durante la pandemia por covid-19 configura sentimientos, experiencias, prácticas y acciones diferenciadas.

After the declaration of a pandemic by the World Health Organization (WHO), on March 19, 2020 the Social, Preventive and Mandatory Isolation measure (Aislamiento Social, Preventivo y Obligatorio, Aspo) was established across Argentina’s national territory. The policy was similar to that of many other countries in the “lockdown” stage of the pandemic in early 2020: isolation at home with the exception of essential activities (healthcare and food provision), social distancing, and mandatory use of face masks. Nevertheless, despite this measure, territorial disparities and entrenched structural inequalities have been the main obstacles to slowing the spread of the new coronavirus ( Hill et al., 2022 ; Bohoslavsky, 2020 ; CCSUC, 2020).

The pandemic has brought profound changes to social dynamics, the economy, and health conditions. In addition to its uneven impact, the pandemic continues to be both an unprecedented event and a “shared” experience for people and societies around the world. However, the fact that it is a collective event does not mean that feelings, experiences, practices, and actions are manifested in the same way for all people or communities. The experience of this crisis is influenced by the way in which people and communities represent and engage with their particular material and social worlds. As Ranger and Slack (1995) argue, the study of epidemics is particularly interesting because, even while they are collective phenomena experienced “in common” across continents and cultures, the ways in which a disease is interpreted and/or constructed makes the symbolic universes of societies more apparent at a given historical moment.

According to Venuleo et al. (2020) , each person interprets the pandemic in terms of specific meanings that are consistent with the symbolic universes that underpin their being and their relationship with their environment. These symbolic frames are defined as systems of implicit generalized assumptions, more or less conscious, that promote interpretations of events, objects, or specific conditions of life ( Salvatore, 2018 ). People’s interpretative categories, therefore, are not simply ways of representing the circumstances and challenges related to the pandemic, but forms of adaptation that enable acting and reacting in certain ways, orient attitudes and behaviors, and shape people’s experiences ( Marinaci et al., 2021 ).

In the literature we find some studies that narrate the experiences of health personnel during the most acute phase of the covid-19 pandemic, providing a view “from the inside” of the health system ( Ramacciotti, 2022 ; Freidin et al., 2021 ; Marinaci, Venuleo, Savarese, 2021; Ortiz et al., 2020 ). However, in our article we propose an integrative perspective that allows us to make the experiences of a broader public more visible, which is a complex challenge in times of pandemic, but fundamental as a basis for advancing in the discovery and exploration of the symbolic frames that configure society. In this sense, studies on the experiences of daily life during the pandemic have identified discourses that show tensions in, and reflections on, personal priorities, everyday life, and social and environmental values, among others ( Venuleo et al., 2020 ; Todorova et al., 2021 ). Likewise, transitions emerge: while in the early phase of the pandemic, narratives focused attention on everyday life and the attempt to make sense of change, in subsequent phases the socioeconomic impact of the crisis became more relevant (Marinaci, Venuleo, Saverese, 2021).

At the same time, we consider the pandemic to be a highly dynamic sociopolitical phenomenon that transcends a public health crisis framing. Although politics played a leading role during the early stage of the crisis, as international organizations and national governments were considered the natural leaders, we note that the sociopolitical aspects of daily experiences of the pandemic have rarely been explored in the literature. Many studies, based on a Foucauldian framework, focus on describing the discursive tendencies of the government during the health emergency: the promotion of biomedical and microbiological discourses, the “grammar of contagion” as an oppressive or punishing force, or the use of positivist knowledges – such as epidemiology – to legitimize government efforts ( Basile, 2020 ). But such scholarly interventions not only present a depersonalized portrait of the government (without agents who have the power to make decisions), but also neglect to analyze how public health policies are received by ordinary people.

For these reasons, this article analyzes sentiments, experiences, practices, and actions that underpin the meanings attributed to the covid-19 pandemic. Taking grounded theory as a starting point – as a strategy to generate new knowledge from qualitative data that are systematically and simultaneously investigated, analyzed, and compared – we are interested in identifying and describing interpretive and symbolic frames of the pandemic. To that end, we carried out a case study in the metropolitan area of San Miguel de Tucumán (Argentina). This is a fragmented territory, marked by social, demographic, economic, and health inequalities. These particularities make for a setting that is especially susceptible to covid-19 and the side effects of control measures, with acute impacts on vulnerable populations (Carter, Cordero, 2022; CCSUC, 2020).

## Research setting and methodology

Tucumán is located in the region called Argentina’s Greater North (Norte Grande Argentino), an area comprised of the nine provinces with the lowest indicators for quality of life, education, poverty, and infant mortality in Argentina (Cordero, Cesani, 2021, 2020; Velázquez, 2014 ). It is the smallest province and the most densely populated of the 23 provinces that make up the national territory, not including the Autonomous City of Buenos Aires (Indec, 2010). Our case study is located in Greater San Miguel de Tucumán, which is the fifth largest metropolitan area in the country.

As in other parts of the world, for Tucumán the year 2020 was marked initially by concern and uncertainty regarding the new and unknown virus, its lethality, and modes of contagion. The declarations of social isolation and later those of social distancing were revised as epidemiological information was updated. After the declaration of the Aspo in March, the community spread of the virus in Tucumán was detected in August, and in September there was an increase in accumulated cases that lasted until November, implying an increase in lethal cases, in line with international averages reported by the WHO (MSP, 2021). The year 2020 concluded with the start of the national vaccination campaign in December.

In June 2021 we began our mixed-method study. In an initial qualitative phase, we carried out focus groups and semi-structured interviews in which twenty key informants from various sectors of society participated. Our key informants belonged to the state sector (in social development, health and education), were representatives of small and medium-sized companies, members of religious institutions, community leaders, mass media communicators (radio, newspapers), and leaders of indigenous communities, among others.

Our intention was to capture a diversity of life experiences that narrate the pandemic not only from an individual perspective, but also linked to the communities of our informants. To identify the dimensions of analysis of a complex, dynamic and multidimensional phenomenon such as the pandemic, we look to the grounded theory approach, since it constitutes a methodology focused on the reflection and study of human behavior in the social world. From this holistic perspective, we are interested in contextualizing and understanding the subjective experiences of social actors and their daily lives.

Prior to the meetings, we developed a set of topics to explore, focused on how people and their communities experienced the changes sparked by the pandemic. During the virtual (online) focus groups, we asked the participants to share their experiences and exchange ideas, with the interviewer guiding the conversation towards the thematic areas. In this sense, our approach centered on an “active and methodical listening” (Bourdieu, Wacquant, Dion, 1995) fostering reflexivity, encouraging people to tell their stories, and highlighting what mattered most to them. It is worth noting that participants often expressed gratitude for the opportunity to share their stories, listen to others, and make sense of what was a collective experience. Subsequently, the recordings were transcribed and coded with Atlas.ti qualitative analysis software.

In a second, more quantitative phase in September 2021, we developed and administered a digital survey that circulated through social media (Facebook and Instagram), making it possible to collect data from 701 participants. With a total of fifty closed items, this survey instrument delved into the central themes that emerged from the qualitative phase of the project. For the analysis of the quantitative data, general prevalences of the responses to the evaluated items were calculated. In addition, chi-square tests were applied for the comparisons between groups, being statistically significant those differences where the p-value was less than 0.05, and highly significant when the p-value was less than 0.001.

In addition, an optional, open-ended question was included, regarding lessons learned during the pandemic. This question was answered by 29% of the respondents (n=203), and their statements were also coded in Atlas.ti. These responses were systematically coded and analyzed, to construct discourses, categories, and hypotheses.

The results of the analysis are presented hereafter.

## Personal resilience, family support and social support as shock absorbers

In 2021, as the second year of the pandemic elapsed, the public was more informed about public health measures, and workplaces and educational settings adapted to hybrid modalities – combining face-to-face and remote work –, along with the implementation of “bubble” shifts, through the formation of small teams, to minimize the possibility of infection and to offer some workplace stability, and with dynamic adjustments in the restrictions as epidemiological conditions changed. In March, the second wave of covid-19 began in the country, a moment that coincided with the reopening of educational establishments. During the second wave, more than 50,000 deaths were reported, along with a decrease in lethality, and 57% of Argentines received at least one dose of vaccine (MSP, 2021).

Our analysis in Tucumán came after the most uncertain months of 2020, at a stage when people and communities were dynamically and continuously adjusting, demonstrating an attitude of “moving forward” as a reflection of resilience. Of the respondents to our online survey, 48.9% were women, the age range of 29 to 39 years predominated (25.2%), and more than half of the participants completed a secondary (high school) education (57.5%). With respect to employment, 23.8% were independent workers, 18.3% were represented by those with employment in the public sector, 17.8% corresponded to those in the private sector, while a small percentage consisted of retired people ( Table 1 ). We were interested in knowing how the participants were affected at that time of the pandemic. Among those surveyed, 43.1% indicated that they had lost their ability to save money, 42% reported food insecurity, and 24% had lost their job. At the same time, 49.2% indicated that people they knew were sickened and/or died from covid-19, while 25.1% indicated that members of their family were similarly affected.

**Table 1:** : Demographic characteristics of study participants, Tucumán, Argentina, 2021

Variables	Overall (n=701)	
	n	%
**Gender**		
Male	357	50.9
Female	343	48.9
**Age**		
18-28 years	224	32.0
29-39 years	177	25.2
40-49 years	166	23.7
50-60 years	134	19.1
**Education**		
No studies	10	1.4
Primary, complete	103	14.7
Secondary, complete	403	57.5
College/University complete	167	23.8
Graduate or higher	18	2.6
**Current job situation**		
Independent worker	167	23.8
Employee (public sector)	128	18.3
Employee (private sector)	125	17.8
Student	120	17.1
Unemployed	107	15.3
Housewife	48	6.8
Retired	6	0.9

Source: Elaboration based on own data (2021).

Although the first months of the pandemic evoked memories of fear and uncertainty, at the time of our analysis there was a general response of adjustment and positive coping, with the pandemic assessed as a personal challenge, not just a social and collective crisis. This development is reflected in the discourses that capture the ability to adapt, self-confidence, a positive attitude to face challenges, and the management of uncertainty. Coping is a constant reciprocal interaction between the environment and people in their routines, where decisions are made that will give rise to certain interactions. Coping strategies are individual responses that mediate in a stressful situation and regulate people’s responses ( Mella-Morambuena et al., 2020 ). The statements of our informants show a prevailing disposition to adapt, to focus actively on solving problems, and to seek ways to support and care for others.

With the passing of these months ... we have not lost our fear, but we have gained courage, and we have also healed many scars from many comrades lost in the line of battle. I think it has strengthened us as professionals, as a society... (Healthcare worker, Focus Group).

... people need to be positive always and not allow themselves to be overcome by the fear of getting sick or dying, because if they think that way they will become depressed and feel bad... (Survey 142, female, 58 years old).

Our quantitative analysis of the surveys is consistent, finding that 81% of respondents used their creativity to solve problems, 74% indicated that they learned to live with the uncertainty brought by the pandemic, and 64% expressed that this experience increased confidence in their ability to solve problems in their personal lives, while 58% said they were able to “move ahead” with a more positive attitude. Respondents generally recognized and capitalized on the learning and skills they had developed, discovering and valuing their own capacities to overcome the problems and challenges that the pandemic emergency, and the measures to contain it, were generating.

Meanwhile, there was widespread use of expressions of religious faith and spirituality that articulate positive feelings, solidarity, optimism towards the future, and the ability to cope with daily life and events beyond one’s control. Survey respondents used the expression “love thy neighbor” as the basis of solidarity, not only as an act of devotion, but also with a prescriptive character, as a social norm. Pargament, Koenig and Pérez (2000) suggest that, in order to face the stressful aspects of life, religious coping uses behaviors such as prayer, confession, the search for spiritual support, and the acceptance of circumstances as representative of the will of God. Studies have shown that spirituality and religion can help people recover from difficult situations, generating relief, hope, meaning, and a sense of value (Alvarado-Diaz, Pagan-Torres, 2021; Van Hook, 2016). From a functionalist perspective, in our analysis, religious faith or spirituality emerges as a resource that allows one to confront, and make sense of, the uncertainty brought on by the pandemic and its control measures, and offers hope for dealing with future challenges. In the region of Northwest Argentina, in general, and the province of Tucumán, in particular, there is a marked influence of religion in everyday life, the cultural landscape, and local history, that allows us to make sense of these sentiments (Ceil, 2019; Martínez, 2010 ). Although there is a wide debate about spirituality or religiosity in circumstances of community crisis or traumatic personal situations, in our analysis these expressions reveal a positive religious coping in relation to the experiences and reflections they describe.

... I consider myself a gladiator of this moment, reborn from the ashes. Hold on to your closest connections. Live the day-to-day. Hug, kiss, and have conversations about things that happen. The uncertainty of life is constant today, and it will be difficult to adjust to the new economic situation in general. May God and the Virgin help us. Thank you (Survey 145, male, 49 years old).

The pandemic only strengthened my confidence and faith to cope with every situation that life throws our way. I care for myself, I care for you (Survey 73, female, 47 years old).

The loss of two people very close to me due to covid taught us that we may be here today, but only God and the Virgin know [what happens] tomorrow (Survey 74, female, 59 years old).

Hopefully the next pandemic will be one of love for your neighbor (Survey 296, female, 25 years old).

In our analysis, people speak eloquently of giving meaning to the pandemic experience and to their own lives, by actively caring for others and seeking to solve problems in their families and communities. In this self-reflection, propositions of responsibility and aid towards others emerged, revealing an ethic of care, especially at the level of intimate ties of affection and trust (toward family, friends, and neighbors). Thus, family and social support networks represent resources that also explain how people have “moved forward” in their interaction with others.

When the pandemic began, it obviously produced many mixed feelings, because sometimes it was scary to think about how to move forward, what will happen to us (Community member, Interview).

So, there was a lot of fear. But not simple fear, [rather] a terrifying fear, things like, how am I going to support the family, how am I going to repay a debt, how am I going to pay this loan or this credit that I took out, all of this really affected us a lot (Community member, Interview).

This ethic of care is also expressed among those who carried out activities that required daily interactions with groups and where the community, to some extent, is perceived as dependent on the activity, or it is understood that ruptures or discontinuities in community work would be detrimental. Such was the case for teachers, managers of soup kitchens, and members of religious communities, among others. People in these circumstances alluded to their ingenuity and their ability to adapt, reinvent workplace dynamics, and maintain activities to fulfill “their duty” to others.

I can tell you that in Education you have no choice but to retrain ... because it’s not like you could lock up the Ministry, leave, and wait for this to situation to pass, do you understand? In other words, we had to restructure, it had to be changed, we had to adapt and continue, because we never stopped teaching. Everything was readapted and we carried on (Educational sector representative, Focus Group).

We had to readapt ourselves and from the religious aspect, people really need support and space. I think that the demands that people make of us on this point are very strong (Parish priest and community leader, Focus Group).

Additionally, the narratives of the pandemic reveal how priorities are identified and redefined, in personal lives and in relationship to others, which leads to the opportunity to give new meaning to one’s life and revalue intimate ties, especially with family. Such reflections on the relationship of subjects with their close, everyday surroundings, as well as the introspective view of personal resilience, aligns with what has been reported in studies of crisis situations. For example, a cross-cultural analysis of the production of meaning during the pandemic notes a convergence in discourses that value family and close and intimate ties, and that reflect on developing more connected and meaningful relationships after this health emergency ( Todorova et al., 2021 ). Likewise, different studies argue that having meaning and purpose in life may mitigate the negative impacts of crises and improve resilience in the face of adversity (Carter, Cordero, 2022; Todorova et al., 2021 ).

## Solidarity as a sign of these times: the value of social capital for communities in a pandemic

In our analysis, solidarity was a recurring theme. It emerges as a way to constructively face the stress of the pandemic, and solidarity is conceptualized from within a discourse that poses it as an unavoidable duty before an external agent “that we have to face.” Participation in, or commitment to, solidarity actions represents a behavioral manifestation of community social capital where people are actively and collectively involved in seeking a suitable situation for members of society ( Lalot et al., 2021 ).

Social capital entails the set of resources, real and potential, that are linked to the possession of a lasting network of reciprocal relationships (Aldrich, Meyer, 2015), and usually constitutes a protective factor. Research conducted during the pandemic has reported that communities where people tend to meet and interact frequently have coped better with the situation (Carter, Cordero, 2022; Borkowska, Laurence, 2021). The literature distinguishes types of social capital as: (1) bonding social capital, which refers to close ties – especially between family members and within kinship networks; (2) bridging social capital, that is, those less-close ties that exist between social groups; and (3) linking social capital, which refers to the relationship between dissimilar groups, with more explicit emphasis on verticality and power inequalities (Aldrich, Meyer, 2015). Our qualitative analysis revealed family and neighborhood groups as the main resources for managing ties of solidarity.

In our experience we gained a better relationship with family and with neighbors, knowing how to support each other in difficult moments (Survey 18, male, 30 years old).

This pandemic has also brought out a lot of solidarity among us. We have started seeing the needs of others, and to be more empathetic with others, whereas perhaps before we did not, because such a tremendous thing [as the pandemic] has never happened to us, because before we faced more personal situations. Now it is a more generalized situation, so we begin to put ourselves in the place of the other (Community member, Interview).

Confirming these sentiments, in our quantitative analysis of surveys, 44.4% (n=311) reported having participated in initiatives to help those most affected by the pandemic. Such initiatives show how bonding social capital is mobilized to carry out acts of solidarity, where work with families (26%) and neighborhood groups (13%), and, to a lesser extent, neighborhood soup kitchens (8%), churches (8%), schools (7%), government bodies such as municipalities and communes (6%), and health centers (4%), were most relevant.

To investigate whether participation in solidarity initiatives varied according to people’s experience of covid-19 infection, we distinguish three groups: those “very affected by covid-19,” that is, those who personally experienced an infection and/or the death of close ties – family and/or friends – (25.1%, n=176) ; “affected,” corresponding to the group of participants who reported knowing people who had the disease and/or died as a consequence (32.4%, n=227); and a third group made up of those “not affected” by the disease, where none of the aforementioned situations were experienced (42.5%, n=298). Our results indicate that the percentage of people who participated in solidarity activities was similar across these groups, with no significant differences when comparing the groups of people highly affected, affected, or not affected by the experience of covid-19 (X^2^: 3.682, p: 0.159).

In addition, we found that people involved in solidarity initiatives reported more confidence in their ability to solve problems in their own lives, compared to those who did not participate in solidarity initiatives (X^2^: 58.332, p: 0.001). These results are related to the qualitative analysis, in which those who participated in solidarity initiatives to help those most affected by the pandemic were more likely to express positive feelings, for example:

It was a very valuable experience to be able to help the indigenous peoples of our province. I received a lot of affection from those people who do not have the possibility of having a more comfortable way of life. I learned from them how valuable the effort and support of the people can be (Survey 3, female, 45 years old).

In the pandemic, working with and assisting people with covid-19, I realized how fragile and short life is. And I learned to place more value on the moments that one spends with loved ones! Because you don’t know when it will be the last time you kiss and hug the one you love! (Survey 8, male, 41 years old).

The pandemic taught me to value others more and get more involved in political issues, since everything revolves around them and our future depends on those decisions (Survey 21, female, 33 years old).

Conversely, negative feelings were found more frequently among participants who reported low participation in solidarity initiatives, for example:

Quarantine (not the pandemic) made us worse off socially and economically. Let’s not vote for more socialism, we need freedom (Survey 63, male, 18 years old).

During this pandemic I came to understand that Argentina is far from being a good life project, I want to leave the country, but it is becoming more and more expensive (Survey 2, female, 20 years old).

On the other hand, 51.5% (n=361) indicated the presence of social capital at the neighborhood or community level. In our survey, we found that people who perceived this type of social capital were more likely to participate in solidarity initiatives to help those most affected by the pandemic, compared to those who did not perceive social capital in their neighborhoods (X^2^: 64.560, p: 0.001). Related to these findings, other researchers have pointed out that sharing intense experiences (positive or negative) with members of one’s own group or close groups (family, neighbors, work colleagues) increases cohesion within the group and the sense of shared social identity, and even more so in the face of negative, dysphoric, or traumatic experiences ( Lalot et al., 2021 ).

Delving into the manifestations of supportive actions, we notice different situations. On the one hand, especially in vulnerable groups with habitual experience dealing with crises, the pandemic has mobilized pre-existing solidarity networks and community strategies in places. In a structural context where infrastructure for basic services such as drinking water and drainage is lacking, where poverty affects 40% of the Tucumán population, including 47% of children and adolescents (Indec, 2010), the coronavirus crisis is experienced as one more challenging contingency to overcome amid structural disadvantages. The needs that emerge are daily nourishment, hygiene, accurate information, psychological and spiritual support, support for small and medium-sized businesses and commerce, and help in access to resources provided by the State. In these areas, the sense of community belonging is marked, and mutual influence or interdependence makes it possible to meet the basic needs of families. Communities have tapped into pre-existing social capital to cope with the pandemic.

Because the government or the State gives us a certain amount of merchandise, the basics, and for everything else we make contributions. We put up a certain amount of money per member and that is how we get the cooking done (Community representative, Interview).

Yes, yes, we have all had covid. And well, the community members and neighbors staffed the soup kitchen. And when one would get better, they would come back, and the others who were infected would go (Community representative, Interview).

On the other hand, the pandemic has stimulated the development of social capital, that is, the formation of alliances, groups, and associations as a strategy to make needs and collective demands visible to the State, and to achieve public-private agreements at the level of the Emergency Operating Committee (Comité Operativo de Emergencia, COE). For example, in the development of health protocols for the reopening of “non-essential” small and medium-sized companies or the management of special aid to independent professionals. We propose that the sense of shared identity is developed and strengthened by a common need or objective, which promotes the generation of bridging and linking social capital. In this sense, we consider it relevant to attend to, and deepen the dynamics of, relations among the State, civil society institutions, and citizens during the pandemic, including affiliations at the community level that help to cope with the crisis.

Through the Chamber [of Commerce], agreements were made with the municipalities, or they speak with councilors, in such a way that produce some ordinance [regulation] that allows certain sectors to open up, to maintain structure and avoid closing their businesses permanently … We look for ways to find solutions for ourselves, as a merchant I am also one of those in that area, so I also have to find my own solutions, just like a large percentage of businesses do. So, it is an everyday job (Businessperson, Interview).

I am in a WhatsApp group of eighty [business] owners. We have obtained approval from the COE, to be able to continue working with certain protocols ... that protocol is currently canceled (Businessperson, Focus Group).

…because we understand that if we collaborate so that people comply with all these [sanitary] regulations it is a benefit, both for society and for ourselves, that we will be able to continue working normally (Businessperson, Company leader, Interview).

In conclusion, we note a common perception that the solutions to the problems posed by the crisis require collective action, which is activated through pre-existing networks or promotes the generation of groups that share interests and objectives. These statements show that the problems that stem from the pandemic have a community-scale scope, with health, economic, and social impacts. Thus, there is an active search for solutions that consider this multiplicity of effects, with complex and flexible political thinking, clearly overcoming the dichotomy of public health *versus* the economy. These forms of interpretation and flexible thinking are relevant as they promote the process of learning from experience ( Todorova et al., 2021 ). The literature explains that, in general, people attribute meanings to their experiences that respond to rigid and polarized ways of thinking, typically organized by oppositions (bad/good, friend/enemy, pleasure/displeasure), while it is less common to find interpretations capable of capturing different kinds of experience and producing differentiated meanings. The latter correspond to more flexible ways of thinking, which then promote learning ( Todorova et al., 2021 ).

## The State: differentiated assessments of health, education and political leaders

During the first months of 2020, official communication was very frequent, with the increased prominence of the president, aligned with provincial governors and officials from different ministries, especially those in the health field ( Klobovs, 2020 ). The policies implemented from within a “State-centric” strategy entailed the vertical coordination of measures designed by the central government and coordinated with the provinces (Azerrat, Ratto, Fantozzi, 2021). In our analysis, the State was recognized as the natural leader during the pandemic, taking on the role of arbiter between interests of the public and private sectors.

The participants in our study manifest a complex view of the role of the State in the management of the pandemic. They identify resource and infrastructure limitations that condition the actions of the different sectors that make up the state apparatus. In this sense, during the pandemic and related control measures, they make differentiated assessments of the organisms that make up the State, according to the repercussions on their daily lives.

There is a positive view of health professionals, as 68% of those surveyed agreed that they did a good job during the pandemic. Survey results are linked to the discourses analyzed qualitatively, which emphasize the vocation of service and the humanity of health professionals, while respondents point to the lack of resources and infrastructure as the causes of failure in the sector during the pandemic.

... despite their abandonment by the government, health personnel were able to face an invisible and deadly enemy, and this also had repercussions on a personal level for every employee, since we had to sacrifice many things so that our families could count on us to provide for them every day (Survey 71, male, 35 years old).

Social media often spread misinformation, [and] the government does not educate the public correctly in the face of a health crisis. Health personnel receive the worst possible labor treatment, while the public expects government support to live on, paid for by those of us who contribute to society. The Education sector suffers many limitations and academic resources were very limited in the face of this challenge that teachers faced with their students (Survey 79, female, 23 years old).

The health sector is very neglected, when it is supposed to be the best prepared in terms of resources, during the pandemic there was a notable lack of empathy towards the [health] professional and the patient on the part of the government, the Ministry of Health, and those in charge (Survey 159, female, 37 years old).

My opinion is that this context [the pandemic] allowed us to see the shortcomings of the health system, it came to light how poorly prepared the health system is in terms of infrastructure, material resources, or supplies. However, it is worth noting the impeccable performance of human resources (doctors, nurses, etc.). It is important to remember the human losses, in order to improve the conditions of the health system in the future (Survey 202, female, 23 years old).

Regarding the education sector, 37% of survey respondents approved of its performance during the pandemic: the situation of educational institutions being closed during the first year of the pandemic, and even during the first half of 2021, was described as problematic. At the same time, our informants emphasized the attitude of teachers who confronted a lack of resources in schools and the demands generated by virtual learning. Interviewees also recognized the challenge of the digital divide. These concerns are well-founded, since half of the households in the San Miguel de Tucumán metro area withstood the challenge of virtual learning without having a computer, and 84% of households accessed the Internet via cell phones (Indec, 2020).

They should also aim to improve education, teachers in the pandemic did their job as best they could, but they are also poorly paid and do not have the necessary resources to offer virtual classes, and in many student homes there is no possibility of connecting [online] to learn (Survey 135, female, 43 years old).

The pandemic tested the creativity of teachers and parents to help children, but unfortunately, there were also inequalities in the right to receive an education. Only those who had a cell phone could learn ... (Survey 201, female, 56 years old).

Finally, there was a low level of approval for political leaders, as only 22% (n=156) of survey respondents agreed that they were capable of leading and managing during the pandemic.

In sum, with the evolution of the pandemic and control measures, people made differentiated judgments about the sectors that comprise the State. Their evaluations show flexible ways of thinking and interpretation, identifying different determinants of State performance depending on the sector. The health sector – especially its human resources – was rated the highest, and political leadership, the lowest.

## Pandemic experience and political sentiment: from honeymoon to disenchantment

During the first months of the pandemic, there was wide recognition of the State’s leadership role, and the level of citizen satisfaction with the president and the national government was high; this positive perception of Argentine politics was evident in the high level of agreement and compliance with public health measures (Rodríguez Varela, Carbonetti, 2021; Klobovs, 2020 ). Employing paternalistic metaphors, political leaders represented the capacity needed to guarantee safety, establish the law, and enforce it ( Quiroga et al., 2020 ). This manifestation of trust and support for government measures is consistent with what has happened in many democratic societies, as the pandemic situation triggered feelings of national unity or solidarity (Hegewald, Schraff, 2022; Devine et al., 2021 ). Many studies indicate that specific events, involving threats or disasters on an international scale, lead to increased support for political leaders by citizens (Hegewald, Schraff, 2022).

By the time our study was carried out, the perception of political legitimacy had changed. As discussed in the previous section, in our quantitative analysis we found that only 22% of survey participants had a positive opinion of the government management of the pandemic.

**Figure 1:**
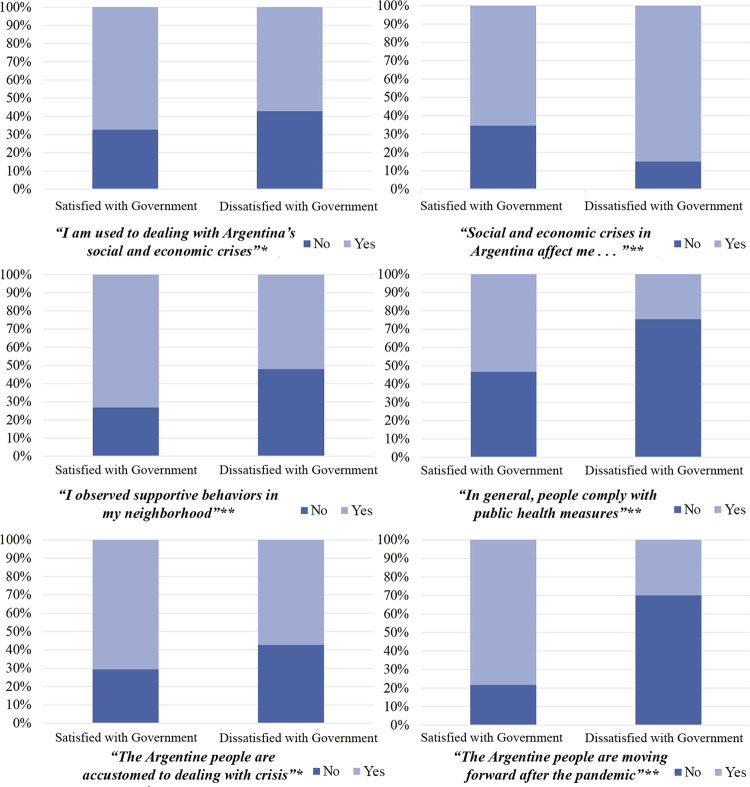
: Charts of differences in individual and collective experiences, according to satisfaction or dissatisfaction with government performance, Tucumán, Argentina, 2021. Statistical differences between groups are indicated with * when p is less than 0.05 (significant), and with ** when p is less than 0.001 (very significant). (Source: Elaboration based on own data, 2021)

To examine this outcome more closely, we compare attitudes between those who expressed support for the government’s management and those who indicated indifference or dissatisfaction. As indicated in Figure 1 , there are significant differences between groups, and we found that those who objected to the government’s actions were more likely to report adverse experiences during the pandemic, compared to people who supported the government. For example, more than 80% of the participants with a negative view of the government’s management agreed with the statement, “social and economic crises in Argentina affect me and do not allow me to make progress.” Likewise, this group was significantly less likely to observe solidarity behaviors to help the most affected in their community, as compared to those who supported the government’s actions. As far as collective perceptions, those who had negative views of the government expressed themselves negatively towards the statements “the Argentine people are used to dealing with social and economic crises” and “in this pandemic situation, the Argentine people are moving forward.”

**Figure 2:**
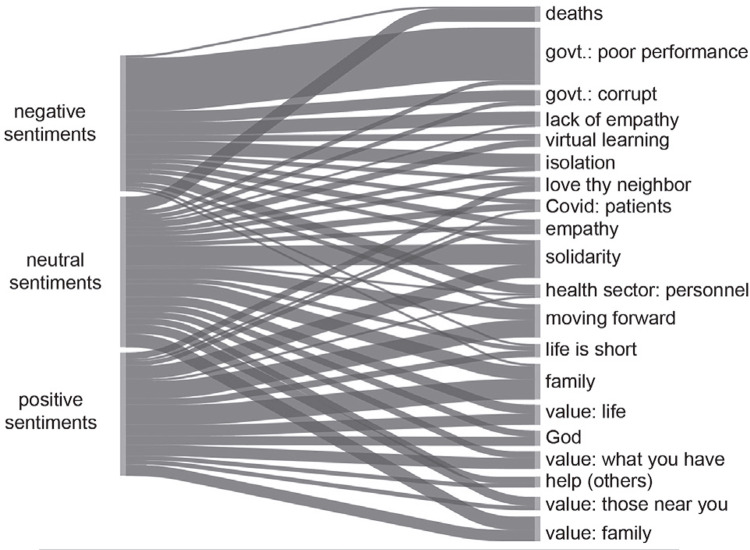
: Lessons learned during the pandemic and political sentiments in Tucumán, Argentina, 2021. This figure shows a “Sankey diagram” from analysis with Atlas.ti software. The relationship between thematic codes is indicated. The results of a cross-tabulation between variables are displayed. Line thickness varies according to the number of phrases where the codes coincide. (Source: Elaboration from own data, 2021)

Next, we analyze the open-ended response to the survey related to lessons learned from the pandemic. As shown in Figure 2 , these statements were classified as expressing negative, neutral, or positive experiences or feelings. It is significant that 18% (n=71) of the people who expressed negative feelings alluded to politics, while among those who expressed positive feelings, politics was only barely mentioned, by only one person (2%). Statements with positive sentiments tended to mention family. In other words, those who shared positive aspects of the pandemic experience highlighted the formation or consolidation of ties with family members and with their social milieu (neighbors, friends, colleagues, etc.), while statements that express discontent are more likely to mention politics.

As the pandemic situation became normalized, this negative evaluation of the government is somewhat expected, given that citizens could develop more careful assessments of the government’s performance as a mediator of interests and as decision-maker (Hegewald, Schraff, 2022). In the context of deep structural inequalities in our study area, government action generated a wide range of challenges – which include the restriction of social contact, the closure of businesses, the loss of income and/or employment, even the impossibility of guaranteeing food security and sustaining livelihoods – placing the concrete and direct personal costs of public health measures in tension with the more abstract and long-term notion of the public good.

Moreover, participants in our study describe negative feelings of disappointment and distrust in response to well-publicized events in Argentina, in which officials violated regulations or allowed preferential access to vaccines. Among these situations, the celebration of the birthday of the First Lady in the Presidential Residence located in the town of Olivos (Buenos Aires province) during the first months of the Aspo (known as the “Fiesta de Olivos”) and the “vaccinations for VIPs” – when officials and associates of politicians were given early access to vaccines, despite not being members of the prioritized groups – were repeatedly mentioned.

Our interviewees mentioned the apathy of political leaders and the abandonment by the State. It is worth noting that these negative discourses on politics evoke feelings of mistrust and disappointment, as well as questions about policy decisions.

For a long time and with the succession of different governments, we live in constant uncertainty, mainly [we are] talking about economic policies that affect our pocketbook. The pandemic exposes the lack of clarity of public, social, economic, health, cultural, education policies, etc., of this government. It leaves us with greater uncertainty than we are used to and feeling that it is increasingly difficult to believe, grow, and undertake projects in Argentina. As an entrepreneur and someone who is passionate about politics, I feel totally excluded from policies that would help independent people/entrepreneurs who do not receive any kind of benefits [from the state] (Survey 17, female, 32 years old).

It seems to me that the government did not make appropriate decisions about [pandemic] restrictions. Because they made restrictions, on the one hand, but, nevertheless, today in political acts there were no restrictive measures in place ... in a political act they did not even consider the pandemic (Survey 12, female, 29 years old).

The antipathy shown by political leaders, such as the party in Olivos and the vaccinations for VIPs, was very sad (Survey 56, female, 54 years old).

I learned that the State only helps slackers ( *vagos* ) and politicians. And I learned that even though I may love Argentina, I have to leave as soon as possible (Survey 39, male, 20 years old).

I could tell you how sad it was to go through those stages [of the pandemic] with the total abandonment by the State, people did not offer work and did not allow you to enter their homes for fear of contagion, on some occasions I STOLE to feed my children (Survey 90, male, 57 years; emphasis in the original).

The worst experience was seeing how the current government sneered at us by stealing the vaccines, and at the same time they [political leaders] did everything that the people were forbidden to do… (Survey 199, male, 47 years old).

At that point in the pandemic, the idea of “the public” had become divided, politicians were no longer united with the people, and a general feeling of disappointment ensued in the face of violated trust. As Reicher and Bauld (2021) suggest, breaking public trust has rapid consequences and is difficult to restore, as confirmed by similar events that occurred in other countries during the pandemic, revealing weak legitimacy in the exercise of a high-ranking positions in the management of public policy ( Bermúdez-Tapia, 2021 ). In this regard, Rodríguez-Pérez (2020) analyzed the moral standards and civil norms that govern society in times of pandemic, and observed a shift from an individualized sense of compliance with the norms, towards loyalty and respect towards the community. While norms are determinants of social behavior, the transgression of the regulations in force during the pandemic by political leaders themselves, not only violated fundamental notions of care, but also showed disrespect towards the community. Our respondents show a deep ethic of social justice that calls into question the existence of different rules for citizens and political leaders.

## Final considerations

Like any other disease, cholera has in itself no meaning: it is only a microorganism. It acquires meaning and significance from its human context, from the ways in which it infiltrates the lives of the people, from the reactions it provokes, and from the manner in which it gives expression to cultural and political values ( Arnold, 1986 , p.151).

As we proposed at the beginning of this article, the covid-19 crisis is a highly dynamic phenomenon that transcends framing as a public health crisis, as it has affected economic and social aspects of the daily life of individuals and communities. Through a grounded theory approach, this article has made it possible to elucidate feelings, experiences, practices, and actions that underlie the meanings attributed to the covid-19 pandemic in Tucumán (Argentina) during 2020-2021. This study provides testimonies and articulates interpretations on the role of the different actors that make up a community, articulating symbolic universes during the covid-19 pandemic.

Our analysis, using mixed methods, explores and describes the way that individuals and communities have signified a particular period, when the pandemic had not ended but had somehow been integrated into daily life. In this sense, the results reveal a transition from an exceptional type of crisis (the pandemic) to a crisis – which we could describe as more typical in Argentina – of a political and economic nature.

The historiography of epidemics reveals ideas and ways of thinking particular to societies of a given historical moment, which configure specific symbolic frames. The intellectual and social responses to, and explanations of, epidemics take different forms in different social, cultural, and political contexts (Ranger, Slack, 1995).

From this perspective, we propose an interesting dichotomy for analytical purposes, resulting from the analysis of the perspectives of participants in our study: we argue that two main symbolic frames organize meanings that our informants use to interpret the pandemic experience.

On the one hand, a “personal” symbolic frame, by which people, through deep reflection, have faced the crisis, finding meaning and managing to signify it through the assessment of their own lives, intimate ties, and the imperative to care for others. From this personal frame of introspection, people have discovered their resilience to face the pandemic: they have put their ingenuity, their ability to adapt, into play; they have actively sought to solve their own problems and collective ones, displaying a genuine willingness to help others and a collective outlook in the search for solutions to the problems and challenges posed by the pandemic.

In this search for meaning, religiosity or spirituality is theme that emerges from our discourse analysis, a topic that few studies on the social response to the pandemic in the Latin American context cover, and one that deserves future research.

In contrast, those who went through the pandemic with a “political” lens expressed negative feelings more frequently. They recognize the leading role of the State as a mediator of interests, but well-publicized events transpired in which politicians undermined the public trust. There is a strong ethic of social justice underlying this perspective, as people question the notion that rights and obligations of citizens are different from those who exercise political power, as exemplified by the so-called “Vacunagate” incident in Peru ( Bermúdez-Tapia, 2021 ) and the “vaccinations for VIPs” in Argentina.

In this regard, we suggest that those who expressed feelings of distrust or disappointment are more likely to apply this political framing to the lessons learned from the pandemic. In contrast, those who signified their experience from a personal frame tend to report better experiences of the pandemic.

However, this analysis has certain limitations that are worthy of mention. On the one hand, our results correspond to a specific moment of the pandemic, which may be different from other stages of the pandemic. On the other hand, although we surveyed a mostly representative sample of participants, it is important to consider that digital resources and Internet service are less accessible for sectors with lower socioeconomic and educational levels. This could produce an underrepresentation of the most vulnerable groups in our data collection methodology carried out during the pandemic.

Still, this study constitutes an original contribution that, through the grounded theory approach, makes visible discourses, perspectives, and subjectivities of social actors, an approach focused on the capacities and resources (material, emotional, affective, spiritual, social) put into action by people and communities to cope with the covid-19 pandemic. Likewise, we highlight as a scientific contribution the solid articulation of theoretical and empirical dialogues that shows experiences of the pandemic beyond a “public health” framing, in a transition stage from the acute phase towards what many viewed, at that moment, as a shift towards a “new normal.” In our opinion, the task of capturing the reflections and experiences of communities, that is, citizens aware of their duties and rights within a community in a pandemic emergency, constitutes an exercise that contributes to progress towards “new emancipatory horizons” for society ( Basile, 2020 ).
